# When Should We Interfere in Threatened Puerperal Convulsions?

**Published:** 1891-06

**Authors:** Everard H. Richardson

**Affiliations:** Atlanta, Ga.


### WHEN SHOULD WE INTERFERE IN THREATENED PUERPERAL CONVULSIONS ?*

BY EVERARD H. RICHARDSON, M. D., ATLANTA, GA.

The proper treatment of threatened cases of convulsions occurring during pregnancy is a subject of supreme importance both to the general practitioner of medicine and to the *accoucheur*. No subject in the domain of medicine is of greater interest to the human family than this grave condition complicating pregnancy, and generally supposed to arise from albuminuria and uræmic poisoning. (All other forms of convulsions are excluded from consideration in this article.) It is not the purpose of the writer to enter into the etiology or pathology of puerperal eclampsia. Its treatment alone will concern us ; and in its consideration we shall speak from the standpoint of the clinician rather than the theorist, promising that facts, in contradistinction to theories, shall dominate in the exposition of the subject. If, in this thesis attempting to set forth the rational and correct treatment for the management of the class of cases under survey, the writer should appear arbitrary or dogmatic, my apology is that I have very positive and absolute convictions on the subject ; and I contend that upon a subject of such vast concern to our race, where the issues of life and death are to be decided so quickly, every one called upon to confront and solve a condition so grave should have crystallized convictions and matured opinions, that he may act, and act promptly, in interest of his patient.

---

*Read Before the Medical Association of Georgia at its Forty-second Annual Session, Augusta, Georgia, April, 1891.

For purposes of illustration the following hypothetical case is presented, which I trust, will be found true to nature : The patient tells us that she has suffered for some days with a severe headache, giddiness, confusion of ideas, restlessness, very nervous and easily fretted. She says that her headache is insufferable, that she has optical illusions, unnatural sounds in ears, and at times has spells of blindness with difficult articulation. Hypocondria and gloomy forebodings of evil are marked. The above are the more prominent subjective symptoms usually present. Coupled with them the objective phenomena observed are, a flushed face, injected conjunctiva, an unnatural stare from eyes; increased heat of skin may or may not be present ; nausea and vomiting, a variable degree of œdema of lower and upper extremeties, extending in some instances to the face, may exist. With this group of symptoms albumen is generally found present in the urine, and the secretion of urine is scant. Under the microscope renal epithelium, casts, blood corpuscles and fibrin cylinders are also observed. The patient presenting these symptoms is usually in the latter half of pregnancy. What then is the duty of the physician when called upon to treat a patient thus suffering, and presenting these symptoms ? Is he justified in wasting precious time by going through the catalogue of remedies supposed to relieve the cause of the symptoms present and prevent the advent of convulsions ? Every moment lost in an expectant or tentative treatment enhances the chances for the supervention of convulsions, which subject the physician and friends to confront the most terrible ordeal and witness the saddest spectacle incident to human life—a mother writhing in convulsions. In the vast majority of such cases *presenting these symptoms* medical treatment is impotent and futile in preventing an outbreak of convulsions. These symptoms show unmistakably profound uræmic poisoning of the nerve centres, and an explosion in the form of terrific convulsions is liable to occur at any moment. The life of the patient is seriously imperilled ; the malady and its attendant results so seriously threatened is grave, terrific, and like an avalanche in character. I am confident that here an expectant and vacillating policy on the part of the physician will inevitably result in the loss of very many valuable lives that might otherwise be saved, and should not for a moment be tolerated by an enlightened profession. I am firm in the conclusion that it is now a very grave responsibility for the attending physician, the guardian of a human life, to fold his hands and content himself by instituting an expectant plan of treatment, hoping with any degree of confidence to avert by any therapeutic agencies now known to the profession, an onslaught of eclampsia. Treatment in all such cases, to be efficient or promising satisfactory results, must be not only active and prompt, but radical and heroic. The few instances in the past where I have voluntarily placed myself in this attitude, I look back upon with feelings akin tothe most exquisite martyrdom and excruciating torture. The uplifted and falling lash that I so painfully see through retrospective lens admonishes me to declare, with all the earnestness and emphasis which the gravity of the situation demands, that the only adequate treatment, the only one promising satisfactory results, must be to thoroughly empty the uterus by the institution of means necessury to produce artificial premature delivery.

The underlying cause of the above symptoms, I think, are due to uræmic poisoning dependent upon acute Bright's disease of pregnancy ; and of this malady the late Carl Braun, of Vienna, says "complete cure is rarely obtained during pregnancy, because the cause of it, the obstruction of the venous circulating in the kidneys, is not easy of removal." But be this as it may, without equivocation or reference to authority on the subject, I wish to give my own views as to the importance in all such cases, *with the symptoms* just narrated, of at once instituting means to terminate the gestation as speedily as possible.

The best means of accomplishing this is by the introduction of an elastic bougie or catheter, under aseptic precautions, into the uterus, supplemented at the proper time by the use of Barnes' uterine dilators. However, *pari passu* with our efforts to accomplish delivery, it is important to get free catharsis from the bowels of patient by elaterinum, calomel, croton oil or hydragogue cathartics, as the emergency of the case may demand. Other measures of treatment are important.

If the symptoms are very urgent, the patient of full habit and nothing to contraindicate it, one general venesection is to be recommended ; this relieves promptly both vascular tension of the brain and kidneys and the toxic influence of the *materies morbi* of the blood, and twenty to thirty grains chloral hydrate is advisable. The patient's room is to be darkened, and she must either be kept under the influence of the chloral, or she must be given chloroform by inhalation during the pains. It is customary to recommend the administration of bromide of potassium in such cases. But I desire to say that there is but one form of convulsions benefited in the least by this agent, and that is epilepsy. It is of undoubted service in the treatment of epilepsy, but I know of no other disease in which its administration is of the slightest advantage. The chloral hydrate in decided doses is not second in importance to even chloroform ; and I strongly recommend the importance of its administration—fifteen grains of this remedy by the mouth every twenty or thirty minutes, or thirty to forty grains per rectum every few hours, until the system is brought under its influence, is greatly to be desired in preventing and controlling convulsions. After the uterus has been well dilated, and if the head does not make sufficient progress, there is no objection to the use of forceps. The important desideratum in the management of all such cases is to deliver as speedily as possible, compatible with safety, using the usual auxiliary means to expedite delivery.

The testimony of Prof. Braun is that "the fits completely cease after evacuation of the uterus in 37 per cent. of cases, become weaker in 31 per cent., and in 32 per cent. only continue of the same severity."

According to my own observation and experience, where the uterus has been emptied, the convulsions have never recurred, except in a very mild form, and I have never known a woman to die from convulsions after delivery had been effected. For purposes of further illustration I will mention the case of Mrs. A.

Was called in consultation with Dr. B. F. Wright, of Polk county. Mrs. A. was in labor at about eighth month of pregnancy ; had had a convultation, but was quiet and asleep when I arrived. Head was well advanced, and I suggested the propriety of using forceps, and delivering as early as possible. Dr. W. insisted on waiting, as he thought child would soon be born. I yielded to him and waited, but only to see the development of hard convulsions in a short time. At once I bled the patient to the extent of twelve to fifteen ounces, and chloroform by inhalation was then administered; while she was still having convulsions I introduced forceps and soon delivered her, to my astonishment, of a living child. Before she came from under the anæsthetic, by *Crede's* method the placenta was delivered. She was washed and placed in bed. As soon as she came from under the influence of chloroform the convulsions returned. Chloroform by inhalation was again resumed. We stood over her till 12 o'clock at night, keeping her under its influence. At this hour we retired, leaving two nurses with instructions that whenever the patient was not asleep and *moved* to administer more chloroform by inhalation. By morning we had the gratification of finding the mother rational and free from danger. Both she and child did well.

Do you ask me to be more explicit and say how soon do I recommend the induction of premature labor in the albuminuria of pregnancy ? To this I reply that in all cases of pregnancy, whenever albumen in the urine is persistently found in large quantities, with or without the presence of any variety of casts, and not yielding promptly to treatment, *whenever decided symptoms* of profound uræmia appear and *continue unabated, then I unqualifiedly* recommend and advise as the safest course to be pursued in the interest of the mother the induction of premature labor. In the face of the facts, the argument that under medical treatment cases of eclampsia may occur and terminate in safe delivery at full term counts for nought when it is remembered that these are exceptional cases and not the rule. In more than fifty per cent. of the cases the lives of the children are sacrified as a result of the circulation of urea in the maternal blood, and with the restrictions here mentioned the question of preserving the life of the fœtus should not be taken into consideration. But the question of superlative importance and paramount to that of all others is, by what method of treatment can we save the greatest number of lives of mothers when imperilled by the malady so much dreaded by all obstetricians.

My views on the subject have been given as the result of personal experience and observation, and without reference to obtaining views of authorities as disclosed in contemporary medical literature. Satisfied that whatever may at present be the *dictum* of authority on this subject, time, the great crucible of all questions, will demonstrate to the candid seeker of truth the correctness of the views herein enunciated. But please do not misapprehend me, and for a moment fancy that I advocate the interruption of gestation for the acute albuminuria of pregnancy, for I am aware that perhaps in the majority of the mild cases of albuminuria occurring during pregnancy they may be safely carried to the end of utero-gestation. For the albuminuria of such cases remedies must be directed to the relief of the kidney insufficiency, and the list of therapeutic agents that have been used with varying degrees of success is a long one. If casts are present in the urine the patient should be confined to bed, and placed upon a milk diet. Benzoic acid, lemon juice, tartaric acid, digitalis, acetate of potassium, coupled with diaphoretics and aperients have all been used and sanctioned by high authority. Dr. H. V. M. Miller of this city has for a number of years treated the albuminuria accompanying pregnancy by the internal use of chloroform, giving from ten to twenty drops every six or eight hours according to the urgency of the symptoms. His own testimony and that of a large number of his followers in this section is unbroken in praise of the efficacy of this method of treatment, the disappearance of the albumen and casts from the urine during treatment attesting the virtue of the treatment. Dr. A. W. Griggs, of West Point, Ga., has also long been an ardent advocate of the chloroform treatment (internally administered) for the acute albuminuria of pregnancy. In chronic Bright's disease of women the subjects should never be permitted to become pregnant. Where pregnancy has occurred in such subjects abortion or premature labor should at once be produced without hesitancy.

But in the cases of albuminuria that have progressed until the nerve centres have become deeply poisoned from the circulation of urea or some poisonous substance in the blood, as shown by prominent head symptoms, then it is that the expectant treatment amounts to playing with the thunderbolt—dallying with human life.

Corroborative of the position taken by the writer in this article, Dr. B. C. Hirst very recently said before the Philadelphia Obstetrical Society that "if mistakes must be made in these cases, and they are inevitable in a situation involved in so much obscurity and doubt as to the outcome, I would prefer occasionally to sacrifice the fœtus unnecessarily rather than occasionally to lose both mother and child by a temporizing policy." And in advocacy of interference he further says, " in any case in which I was in serious doubt as to the course to pursue in the future, I would always decide in favor of terminating pregnancy."

Dr. Edward L. Duer, in discussing the propriety of interference, said, "Where there is an alarming amount of albumen—say about twelve per cent.—examination of the urine should be made every two or three hours, and if the patient fails to respond to the treatment, especially if tube casts be present, I think we should risk the loss of the child rather than jeopardize the life of the child and mother.

ECLAMPSIA.

Upon the subject of the treatment of puerperal convulsions, when they appear there remains but little to be said. In the very beginning of treatment, unless contraindicated on account of great feebleness of the patient, phlebotomy, promptly resorted to, is very greatly to be desired.

Relieve the blood tension of brain, lungs and kidneys, thereby preventing apoplexy of brain and pulmonary œdema, and aiding at the same time the elimination of a certain amount of the *materies morbi* of the blood by the abstraction of from twelve to twenty-five ounces of blood. Next in importance is the administration of hydrate of chloral in very decided doses. Of this agent give thirty grains by the mouth or sixty grains by the rectum, and repeat whenever the patient shows evidence of coming from under its influence.

Until the chloral has been absorbed, when there is a premonition of the advent of convulsions, chloroform, by inhalation, may be given. It is also important to obtain free catharsis by the action of croton oil, elaterinum or calomel. Free diaphoresis by the hot wet pack, hot water bags, hot bottles, etc., is desirable. Veratrum viride I have used hypodermically with excellent results, but its use requires caution, and it is impossible or injudicious to use every good remedy on the same case. After the use of the agents above mentioned I have generally been able to dispense with veratrum in the treatment of puerperal eclampsia. Pilocarpia I allude to only to condemn its use in this class of cases. The use of morphia in the treatment of the eclampsia of pregnancy has been extolled and condemned by authorities of equal merit, and individual experience must decide its value. Rationally I do not think it indicated, and I am sure that I have never used it for the cases under consideration with the slightest benefit to my patients. It is also of supreme importance now to aid nature in her efforts to effect delivery of the child, either by digital dilatation of the uterus or with Barnes, or other dilators. Dilatation of the uterine canal having been accomplished, delivery may be completed by version or the forceps as the practitioner may elect. If during this procedure the mother has been assiduously kept under the combined influence of chloral and chloroform the heroic measures advised have very materially enhanced her chances of recovery.

